# Molecular detection and characterization of *Rickettsia felis*, *R. asembonensis*, and *Yersinia pestis* from peri-domestic fleas in Uganda

**DOI:** 10.1080/20008686.2025.2473159

**Published:** 2025-03-03

**Authors:** Wilfred Eneku, Bernard Erima, Anatoli Byaruhanga Maranda, Nora Cleary Gillian, Gladys Atim, Titus Tugume, Qouilazoni Ukuli Aquino, Hannah Kibuuka, Edison Mworozi, Christina Douglas, Jeffrey Koehler William, Michael von Fricken Emery, Robert Tweyongyere, Fred Wabwire-Mangen, Denis Byarugaba Karuhize

**Affiliations:** a College of Veterinary Medicine, Makerere University, Kampala, Uganda; b Emerging Infectious Disease Department, Makerere University Walter Reed Project, Kampala, Uganda; c Environmental and Global Health, University of Florida, Gainesville, FL, USA; d College of Health Sciences, Makerere University, Kampala, Uganda; e Diagnostic Systems Division, USAMRIID, Fort Detrick, MD, USA; f School of Public Health, Makerere University, Kampala, Uganda

**Keywords:** Siphonaptera, entomology, zoonoses, *Rickettsia*, Uganda

## Abstract

**Background:** Fleas transmit a variety of zoonotic agents whose epidemiology and public health risk remains poorly understood in sub-Saharan Africa, including Uganda particularly outside plague-endemic areas. Common flea-borne zoonotic agents include *Rickettsia felis* and *Yersinia pestis.*.

**Objectives:** The study aimed at detecting and characterising flea-borne pathogens in peridomestic environments in Uganda.

**Methods:** We collected fleas from domestic animals, chickens, rodents, and homestead environments; pooled them by species, collection time, and host species. A total of 172 pools were analyzed for *Y. pestis Pla* gene. Further, 62 pools were tested for *Rickettsia* species *gltA, ompA*, and *htrA* genes by PCR and Sanger sequencing.

**Results:** Five flea species were identified: *C. canis, C. felis, Echidnophaga gallinacea, Pulex irritans*, and *X. cheopis*. Genus, *Ctenocephalides,* accounted for 84.8% of fleas collected, mostly found on dogs and goats. The flea species were found across all districts, year-round, with higher numbers collected in dry seasons than rainy seasons. *Rattus rattus* constituted 74% of rodents captured from human dwellings and was the only rodent species with fleas, where *X. cheopis* was the predominant species. All 172 pools were negative for *Y. pestis*. *Rickettsia* spp. was detected in 29/62 (46.8%) pools by the target genes. Of 25 *htrA* amplicons sequenced, 4% were identified as *R. felis* from *C. canis*, 92% were *R. asembonensis* from multiple flea species, and 4% were identified as *Candidatus* Rickettsia senegalensis.

**Conclusion:**The survey identified high pool detection rate of *Rickettsia* spp. in fleas,suggestingrisk of human exposure and infection. This was the first report of *Rickettsia* spp. in *E. gallinacea* and detection of *Candidatus* R. senegalensis in Uganda.

## Introduction

*Rickettsia felis* is an obligate intracellular gram-negative bacterium in the class alpha-proteobacteria. The *Rickettsia* genus is divided broadly into four groups based on genotyping: the ancestral group, typhus group, spotted fever group, and transitional group to which *R. felis* belongs [1]. However, recently, a five-level grouping of *Rickettsia* based on phylogenetic analysis separates the ancestral grouping into Belli group and Canadensis group, transitional group into SFG II, tickborne *Rickettsia* into SFG I, and and the typhus group [2]. *Rickettsia felis* was first detected in colonized cat fleas, *Ctenocephalides felis*, in 1990s in the US [3]. *R. felis* has been widely regarded as an emerging human pathogen with the detection of DNA and antibodies of *R. felis* in patients with undifferentiated febrile illness being reported across the world [4,5]. Despite limited evidence, *R. felis* has been associated with the occurrence of acute febrile illness in sub-Saharan Africa [6,7]. *R. felis* DNA has been detected in up to 15% of febrile patients from Mali, Senegal, Gabon, and Kenya [8]. Through molecular genetics and surveillance of flea-borne rickettsial agents in fleas, *R. felis*-like organisms, such as *Rickettsia asembonensis*, and *Candidatus* Rickettsia senegalensis have also been detected in various flea species, although their pathogenicity remains unknown [9]. Diagnosis of flea-borne rickettsioses is difficult as they cannot be cultured using conventional bacteriological methods due to their intracellular nature. As a result, they often go undiagnosed due to lack of access to advanced diagnostic techniques, which are often unavailable in resource-limited settings [10].

Plague, caused by *Yersinia pestis*, is a flea-borne bacterial zoonosis that is often fatal if appropriate antibiotic treatment is inadequate or delayed [11]. The majority of human plague cases in recent decades have occurred in Africa [12]. In Uganda, the West Nile region represents the primary epidemiologic foci where 255 human plague cases were reported between 2008 and 2016 [13]. Due to the epidemic nature of the disease, many studies about *Y. pestis* and its epidemiology have been concentrated in this region, neglecting other areas in Uganda.

Fleas, particularly adults, are obligatory hematophagous wingless ectoparasites of higher vertebrates, particularly mammals and birds [14]. Due to their blood feeding habits across a wide host range, fleas are important vectors for pathogens of both medical and veterinary importance [14,15]. The evolutionary success of fleas has enabled them to survive globally in a variety of landscapes and environmental conditions. The most recognized fleas from medical and veterinary perspectives are several pulicids (*Pulex irritans, Xenopsylla cheopis, Ctenocephalides canis, C. felis, Echidnophaga gallinacea*), ceratophyllids (*Nosopsyllus consimilis, Nosopsyllus fasciatus*), and one leptopsyllid (*Leptopsylla segnis*) [14]. The global distribution of these species is a result of their close interaction with humans, livestock, pets, and synanthropic animals (mice and rats). Despite their world-wide distribution, the dissemination of these fleas is not uniform [14]. Instead, they are distributed in patches that are characterized by the host and environmental conditions based on favorability of each given species. This may explain the focal occurrences of the diseases they transmit.

*C. felis* (cat fleas) are hosts and vectors of *R. felis* and *R. asembonensis* [7]. However, the detection of *R. felis* in other arthropods such as other flea species, ticks, and *Anopheles* mosquitoes have been reported [16]. Additionally, *R. asembonensis* has been found in other flea species including *C. canis*, *P. irritans* and a variety of arthropods including ticks [17,18]. Rodent fleas, particularly *X. cheopis* and *Xenopsylla brasiliensis*, are the main vectors of *Y. pestis* [19,20]. However, *C. felis* has also been demonstrated to transmit *Y. pestis* in its early stages of feeding and is a potential vector in endemic areas or hotspots of re-emergence [21]. *C. felis* are indiscriminate feeders that parasitize domestic cats, dogs and other livestock including ruminants. The bird flea, *E. gallinacea*, is characterized as a stationary feeder, and like other fleas, it also parasitizes mammals [14].

In Uganda, the epidemiology of *R. felis* and its role as potential cause of febrile illness has largely been neglected. Furthermore, the flea populations, their preferred hosts, and the pathogens they carry in domestic and peri-domestic areas have been understudied outside plague-endemic foci of the West Nile region of Uganda. In this study, we sought to describe flea–host associations on animals in four regions of Uganda outside the West Nile region, determine the flea index on animals from homesteads, and screen fleas for flea-borne pathogens, specifically *Rickettsia* spp. and *Y. pestis*. This study adds critical epidemiological information on circulating flea-borne pathogens and can be used to improve prevention and control efforts.

## Materials and methods

### Study design and sites

**Figure 1.**
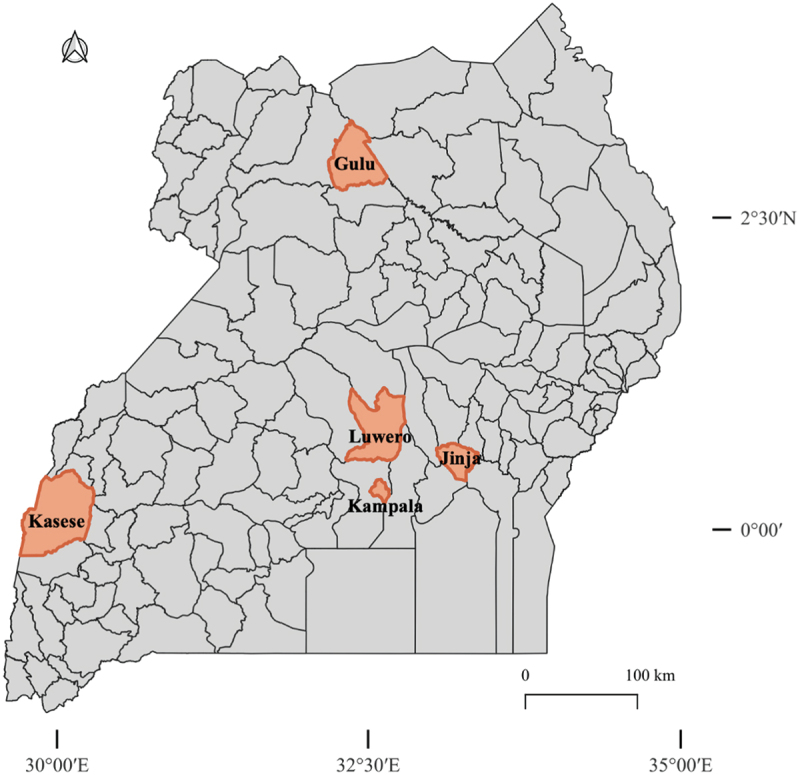
Study districts (shown in orange) for arthropod vector surveillance in Uganda.

This was a cross-sectional study in which we collected fleas in five districts of Uganda, namely Jinja (Eastern Uganda), Kampala (Capital of Uganda), Kasese (Western Uganda), Gulu (Northern Uganda), and Luwero (Central Uganda) (Figure 1) between April 2017 and September 2018. The selected districts are considered major economic hubs in their respective regions and are both geographically and culturally diverse, with high levels of economic heterogeneity. The regions receive bimodal rainfall pattern with short rainfalls from April to June and a second, more reliable wet season from September to November.

### Sample size determination and selection of households for flea collection

Animal owning households were targeted for flea collection. Based on national estimates of households owning animals at 72.8% and using the sample size calculation formula for cross-sectional studies [22], set to a 95% confidence interval and margin of error of 5%, the required sample size for statistical power was 335 households. A total of 360 households, factoring for non-response rates, were targeted in the five districts in the sampling period. In each district, 18 villages were chosen by simple random sampling and three villages for each three-month quarter (dry and wet seasons) were sampled. The list of villages used for selection was based on the Uganda Electoral Commission database and the land conflict mapping tool (www.lcmt.org/uganda). Four animal owning households per village were selected for flea collection with the help of local leaders. Informed consent was obtained from the head of the household. Both food and companion animals were included with no regard to breed, sex or age. Fleas were collected from livestock (cattle, goats, sheep, pigs, rabbits), companion animals (dogs and cats), chickens, rodents, and directly from the environment. When the target collection number was not reached from a homestead, the nearest neighbour(s) was included in sampling.

### Flea collection and pooling

Most mammalian fleas do not attach firmly to hosts during feeding and can easily escape when disturbed, so attempts to restrain and immobilize the fleas were made [14]. Pets with phlegmatic temperament and small ruminants were hand restrained. Aggressive pets were sedated with Xylazine 1 mg/kg (Xyla, Interchemie, Holland) and Ketamine 6 mg/kg (Rotex Medica, Trittau, Germany). Animals were placed on white plastic sheets, and the surface of their fur was sprayed with 70% ethanol to immobilize fleas. After one to four minutes, fleas under the fur emerged to the surface and were brushed onto the white sheet, picked with forceps, and transferred into 70% ethanol for preservation. Fleas from each animal were kept in a labeled separate screw-caped 7 ml Bijou bottles. Rodents were trapped using Sherman traps in houses occupied or frequently visited by people in the selected homesteads. Five traps were set per household and a total of 20 traps in the village in one night. The traps were baited and set in the evening with a mixture of ground peanuts and smoked fish, with captured rodents recovered, transported to laboratory or field station in white aerated gunny bags in the morning. In the laboratory, the gunny bags were sprayed with 70% ethanol to prevent escape of fleas and the rodents were euthanized by inhalation of overdose of halothane (Piramal, Andhra Pradesh, India) as previously described [19]. Traps were transported in white aerated gunny bags to prevent fleas from escaping. The rodent species were identified by morphological characteristics including the length of their body, tail, ear, hind foot, weight [23]. Fleas were collected by combing the fur with a brush on to white gunny bags and placed in separate bottles containing 70% ethanol for preservation as above. The flea index (# of fleas/host examined) for the animals was determined using established methods [24]. Fleas were also collected from earthen floor houses inhabited or frequented by people in the homesteads. We used trays containing water with grease or Vaseline smeared on the sides to prevent fleas from crawling as previously described [9]. Fleas were identified to the species level using published morphologic taxonomic keys under a stereomicroscope [15,25,26]. A total of 14,641 individual fleas were collected and identified. The identified fleas were pooled into 714 sample pools (with sizes ranging from 1 to 281 fleas) according to flea species, host species, collection area and date of collection. When two goats in the same household had *C. canis*, the fleas would be pooled into one pool whereas two flea species on one goat would form two separate pools.

### Homogenization of the flea pools

Flea pools were placed in Eppendorf tubes containing RNA later (Sigma Life Science, Darmstadt, Germany) and disrupted using sterile disposable pestles attached to a motorized grinder (HLD-12, Ryobi, China). The coarse disrupted flea samples were then homogenized by passing them through 20-gauge needles. The homogenates were stored at −80°C until DNA extraction. A total of 172 flea pools consisting of all the 68 rodent flea pools collected and an additional 104 pools collected from pets, livestock, and the environment were tested for *Y pestis*. Each pool consisted of 1–63 fleas. All the rodent flea pools (three pools of *E. gallinacea*, 65 *X. cheopis*) were tested since rodents are reservoirs of *Y. pestis* and their fleas are major vectors for *Y. pestis*. The additional 104 flea pools selected for *Y. pestis* testing comprised the five flea species collected in the five districts and two seasons (dry and rainy seasons). The two seasons correspond to the first two quarters of flea collection (Supplementary File 2, Table 1). The Apr–Jun (rainy) season is a period of low epizootic activity while July–Sept (dry) is a period of the active plague season in the West Nile region of Uganda [19,27]. Of the 172 pools, a subset of 62 pools (353 individual fleas) with pool sizes of 1–28 fleas were selected based on seasons (dry and wet), village representation, and subsequently tested for *Rickettsia* spp.

**Table 1.** PCR description for *rickettsia* spp. and *Y. pestis.*

PCR reaction target						
Species	Gene	PCR type	Primer Name	Primer Sequence	Product size	Annealing temperature
*Rickettsia* spp.	*gltA*	qPCR	CS-F	5-TCGCAAATGTTCACGGTACTTT-3	74bp	60°C, 30s
			CS-R	5-TCGTGCATTTCTTTCCATTGTG-3		
			CSP-P (Probe)	6-FAM-TGCAATAGCAAGAACCGTAGGCTGGATG-BHQ-1-3		
*Rickettsia* spp.	*htrA*	qPCR	R17K128F	5-GGGCGGTATGAAYAAACAAG-3	115bp	60°C, 30s
			R17K238R	5-CCTACACCTACTCCVACAAG-3		
			R17 K202TaqP	6FAM-CCGAATTGAGAACCAAGTAATGC-TAMRA		
*Rickettsia* spp.	*htrA*	PCR	Rr17k 1p	TTTACAAAATTCTAAAAACCAT	539bp	57°C, 60s
			Rr17k 539n	TCAATTCACAACTTGCCATT		
*Rickettsia* spp.	*ompA*	PCR	Rr190k 71p	TGGCGAATATTTCTCCAAAA	650bp	42°C, 35s
			Rr190k 720n	TGCATTTGTATTACCTATTGT		
*Y. pestis*	*Pla*	qPCR	Yper_PLA_F	5-ATGGAGCTTATACCGGAAAC-3	98bp	60°C, 30s
			Yper_PLA_R	5-GCGATACTGGCCTGCAAG-3		
			Yper_PLA_P	6- FAM-TCCCGAAAGGAGTGCGGGTAATAGG- TAMRA		

### DNA extraction and PCR

Total DNA was extracted from all the flea homogenates using the Qiagen DNeasy Blood and Tissue kit (Qiagen, Hilden, Germany), according to the manufacturer’s protocol. Duplicate *Rickettsia* positive whole cell lysate (provided by Walter Reed Army Institute of Research, Silver Spring, MD) and negative controls were included during every batch of DNA extraction. The 62 flea pool DNA samples were screened for flea-borne *Rickettsia* spp. with primers amplifying the 74-bp citrate synthase (*gltA*) gene as previously described (Table 1) [9,28,29]. A second *Rickettsia* genus-specific qPCR amplified a 115-bp segment of the *htrA* gene to confirm the initial PCR results as described previously [30,31]. *R. felis* DNA was used as a positive control and ultrapure water as a negative control. To detect *Y. pestis* from flea pools, qPCR targeting the plasminogen activator gene (*Pla*) was performed [32,33]. *Y. pestis* CSUR P100 strain DNA was used as positive control.

### Sequencing and phylogenetic analysis

A 539 base-pair amplicon for *htrA* and 650 base-pair amplicons for *ompA* gene were amplified as previously described [34] using Platinum Taq (Thermo Fisher Scientific). The PCR products were purified using the QIAquick PCR Purification Kit (Qiagen) according to the manufacturer’s instructions. Purified amplicons from Agarose gel for the *htrA* and *ompA* genes were sequenced on the SeqStudio (Thermo Fisher Scientific) using the BigDye Terminator v3.1 Cycle Sequencing Kit (Thermo Fisher Scientific) according to the manufacturer’s recommendations. Forward and reverse reads were aligned using CLC Genomics Workbench (Qiagen), and a consensus sequence for each gene was generated for BLAST analysis. Sequences of *htrA* and *ompA* rickettsia genes and references from GenBank were imported and aligned in Geneious Prime 2022.11.0.14.1. The sequences were MAFFT aligned and exported to MEGA 10.2.6 [35]. Maximum likelihood trees were created using the Tamura-Nei model with bootstrap iterations set at 1,000.

### Mapping

Descriptive maps for the flea collection sites, flea species, and pathogen location were generated in QGIS 3.28 [36]. The Uganda district shapefiles were accessed at https://data.unhcr.org/en/documents/details/83043.

### Statistical analysis

Flea indices for the samples collected from the animals were calculated as previously described [27]. The pooled positivity rates, minimum infection rates (MIR), and maximum likelihood estimation (MLE) were calculated for collection district and flea species to assess the likelihood of *Rickettsia* spp. detection from the flea pools as described before [37]. MIR was determined by dividing the number of infected pools by the total number of fleas in all the pools and expressed as the number of infected pools per 100 fleas tested assuming that each positive pool only had one positive flea. The maximum-likelihood estimate (MLE) is the infection rate most likely observed given the test results and an assumed probabilistic model, which is a binomial distribution of infected individuals in a positive pool. The CDC’s Mosquito Surveillance Software (https://www.cdc.gov/westnile/resourcepages/mosqSurvSoft.html) was used in Excel to obtain MLE and MIR estimates with their corresponding 95% confidence intervals accounting for individual pool sample size. A Pearson chi-squared test was used to detect any differences between the distributions of outcomes in different groups, with a p-value of <0.05 considered significant. Data were analyzed using STATA software, version 16.1 (StataCorp, College Station, TX).

## Results

### Flea infestation of the hosts and environment

A total of 14,641 individual fleas, comprising five species were collected from livestock, companion animals, rodents, and the environment during the sampling period (Table 2). Rodents and shrews (*n* = 159) belonging to three species were collected from human dwellings in the five districts over the 18-month collection period (7200 trap nights). The trap success rate was low (2.2%). The three rodent species trapped were *Rattus rattus* (118/159; 74.2%), *Mus musculus* (32/159; 20.1%) and *Crocidura* spp. (9/159; 5.7%) (Supplementary File 1, Table 1). Among rodents, fleas were only recovered from *R. rattus* and none from shrews (*Crocidura* spp.) and mice (*M. musculus*). From *R. rattus*, 256 fleas belonging to two species, *X. cheopis* and *E. gallinacea*, were recovered. A total of 119 fleas belonging to three species (*C. canis*- 116, *E. gallinacea*- 2 and *P. irritans*- 1) were collected from 13 domestic rabbits. *E. gallinacea* (9.3% of all fleas collected) was recovered from rodents, cats, dogs, goats, pigs, rabbits, and the environment, and *X. cheopis* was found on cats, dogs, and goats. The predominant fleas, *C. canis* (84.8% of all fleas collected), were collected on all animals except chickens and rodents. *C. felis*, collected from cats constituted 4.1% of all the fleas collected and was the third most abundant flea species identified. Based on the flea index, dogs were the most infested domestic mammal, followed by rabbits and then goats (Table 2). Chicken had a high flea-index though predominantly constituted by one species, *E. gallinacea*. The overall flea index for *X. cheopis* from *R. rattus* was 1.9, which is considerably high. The most flea infested district was Kasese (25.2%), followed by Jinja (20.6%) and the least was Kampala (17.2%). The infestation rate between the districts was statistically significant (χ^*2*^ = 43.04, *df* = 16, *p* < 0.001). However, Gulu had the most diverse flea species, with five species recovered. There were significant differences observed in flea diversity between districts (χ^*2*^ = 42.51, *df* = 16, *p* < 0.001) and seasons (quarters) (χ^*2*^ = 47.64, *df* = 20, *p* < 0.001) of collection (Supplementary File 2, Tables S1–S2).

**Table 2.** Flea index and collection number on animals and the environment in five districts from April 2017–Sept 2018.

	Total number of fleas collected (Number of fleas/host examined)					
Host (Numbers checked)	*C. canis*	*C. felis*	*E. gallinacea*	*P. irritans*	*X. cheopis*	Total
Livestock						
Cattle (3)	31 (Nd)	0 (Nd)	0 (Nd)	0 (Nd)	0 (Nd)	31 (Nd)
Goat (1196)	6286 (5.26)	–	13 (0.01)	–	8 (0.01)	6307 (5.27)
Sheep (12)	86 (Nd)	0 (Nd)	0 (Nd)	0 (Nd)	0 (Nd)	86 (Nd)
Pig (18)	115 (Nd)	0 (Nd)	8 (Nd)	0 (Nd)	0 (Nd)	123 (Nd)
Rabbit (13)	116 (8.92)	–	2 (0.15)	1 (0.08)	–	119 (9.15)
Chicken (8)	–	–	366 (45.75)	–	–	366 (45.75)
Domestic animal						
Dog (343)	5703 (16.66)	–	428 (1.25)	–	23 (0.07)	6154 (17.94)
Cat (122)	3 (0.02)	604 (5.00)	407 (3.34)	–	3 (0.02)	1017 (8.34)
Rodent						
*Rattus rattus* (118)	–	–	32 (0.27)	–	224 (1.90)	256 (2.17)
*Crocidura* spp. (9)	–	–	–	–	–	–
*Mus musculus* (32)	–	–	–	–	–	–
Environment						
Earthen floor house (124)	82 (Nd)	0 (Nd)	100 (Nd)	0 (Nd)	0 (Nd)	182 (Nd)
Total Fleas	12422	604	1356	1	258	14641

Nd: flea index not determined. For cattle, only calves were fully checked because mature cattle were not restrained for a complete examination due to the risk of bloat. Pigs were not entirely searched for fleas because they were not fully restrained by hand. Sheep were not considered in this estimate as it was not practical to comb for all fleas due to variable amounts of wool.

### Infection rates of Rickettsia spp. and Y. pestis in the flea pools from the districts

Pool positivity rates for *Rickettsia* spp. from the 62 flea pools consisting of 353 fleas tested with qPCR for *gltA* and *htrA* gene primers are presented in Table 3. *Rickettsia* spp. was detected in all districts (Supplementary Figure S1) with an overall pooled positivity rate of 48.8% (95% CI: 34.4–59.2).

**Table 3.** Maximum likelihood estimates (MLE) and minimum infection rate (MIR) with corresponding 95% confidence intervals for detection rates of *Rickettsia* spp. in all flea pools.

					MLE			MIR		
District	Flea species	Positive pools (%)		Total fleas	Point	Low	High	Point	Low	High
Gulu	*C. canis*	7/9	(77.8%)	42	30.59	14.25	63.58	16.67	5.40	27.94
	*E. gallinacea*	2/4	(50.0%)	7	29.01	6.11	68.39	28.57	0.00	62.04
	*P. irritans*	0/1	(0%)	1	0.00	0.00	79.35	0.00	–	–
	*X. cheopis*	2/3	(66.7%)	14	16.10	4.25	66.80	14.29	0.00	32.62
	Subtotal	11/17	(64.7%)	64	29.76	16.41	50.50	17.19	7.95	26.43
Kasese	*C. canis*	4/6	(66.7%)	55	11.85	3.99	34.53	7.27	0.41	14.14
	*X. cheopis*	0/3	(0%)	13	0.00	0.00	15.62	0.00	–	–
	Subtotal	4/9	(44.4%)	68	8.33	2.74	21.76	5.88	0.29	11.48
Kampala	*C. canis*	2/4	(50.0%)	32	8.01	1.85	40.69	6.25	0.00	14.64
	*C. felis*	1/1	(100%)	7	–	–	–	14.29	0.00	40.21
	*E. gallinacea*	0/1	(0%)	1	0.00	0.00	79.35	0.00	–	–
	*X. cheopis*	0/5	(0%)	12	0.00	0.00	19.15	0.00	–	–
	Subtotal	3/11	(27.3%)	52	7.98	2.15	23.69	5.77	0.00	12.11
Jinja	*C. canis*	6/7	(85.7%)	53	18.36	9.20	18.36	11.32	2.79	19.85
	*X. cheopis*	1/6	(16.7%)	24	3.85	0.25	3.85	4.17	0.00	12.16
	Subtotal	7/13	(53.8%)	77	12.59	5.97	24.90	9.09	2.67	15.51
Luwero	*C. canis*	4/11	(36.4%)	90	4.65	1.74	10.57	4.44	0.19	8.70
	*X. cheopis*	0/1	(0%)	2	0.00	0.00	54.55	0.00	–	–
	Subtotal	4/12	(33.3%)	92	4.56	1.69	10.36	4.35	0.18	8.52
	Overall total	29/62	(46.8%)	353	11.55	8.15	16.07	8.22	5.35	11.08

Overall, MLE for *Rickettsia* spp. for the districts was 11.5% (95% CI: 8.2–16.1), with the corresponding MIR of 8.2% (95% CI: 5.4–11.1). Gulu district had the highest MLE of 29.8% (95% CI: 16.4–50.5) with a corresponding MIR of 17.2% (95% CI: 7.9–26.4) while Luwero had the lowest MLE of 4.6% (95% CI: 1.7–10.4) with a MIR of 4.3% (95% CI: 0.2–9.0). In general, higher MLE values were obtained in the northern and eastern districts. *Rickettsia*-positive flea pools were from three genera, *Ctenocephalides* (82.8%), *Xenopsylla* (10.3%) and *Echidnophaga* (6.9%). *C. canis* had the highest MLE and MIR across flea species, while other flea species had variable MLEs based on districts of collection (Table 3). None of the 172 flea pools consisting of 2,457 fleas from all five species, amplified for *Y. pestis*.

### Rickettsia spp. identified by nucleotide sequences and phylogenetic analysis

**Figure 2.**
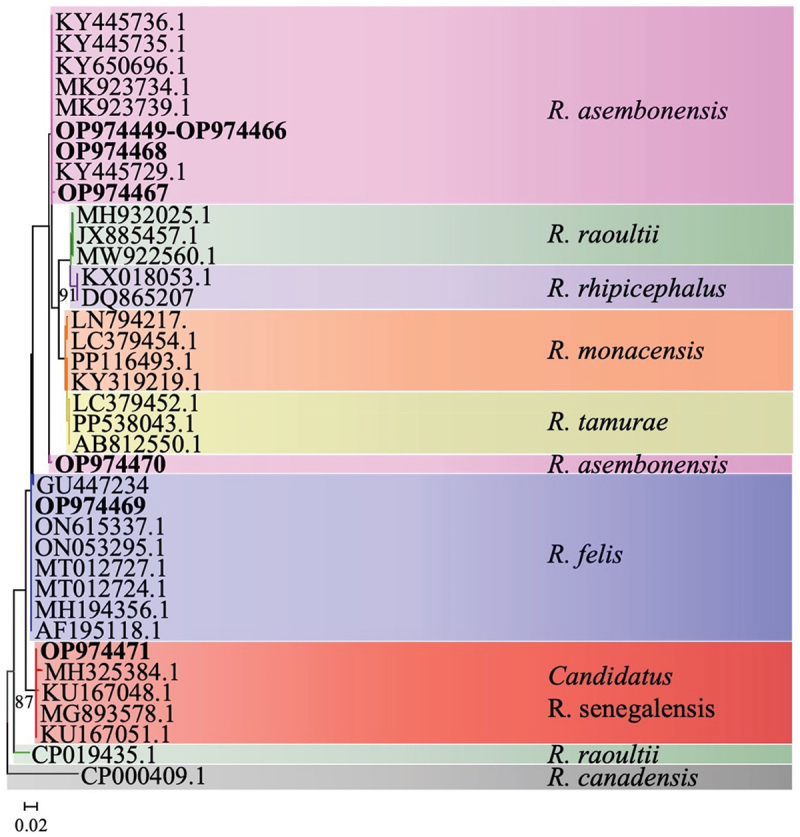
Maximum likelihood tree of the htrA *Rickettsia* spp. gene with sequences ranging from 382 to 451bp with bootstrap iterations set to 1,000. Values less than 70% were excluded from the tree. GenBank numbers in bold were from this study.

Of the 29 flea pools positive for *Rickettsia* spp., 25 pools of purified PCR amplicons were successfully sequenced using the *htrA* genes. The nucleotide sequences obtained from *htrA* and *ompA* were compared to those available on NCBI GenBank database by BLASTn analyses. The summary of the *Rickettsia* spp. identified by sequences and phylogenetic trees are presented in Table 4 and Figure 2. The predominant *Rickettsia* spp. identified was *R. asembonensis* (23/25). *R. asembonensis* was only detected in *C. canis* from multiple animal species (goat, dog, cat, and a rabbit). The comparison of the *htrA* sequence from this study to GenBank sequences revealed that flea pools from Gulu, Jinja, Luwero and Kampala had identical homology with a sequence isolated from *C. canis* (MK923739.1) and *C. felis* (KY650696.1) in Peru and *C. felis* from Brazil (KY445736.1). A sequence of *R. felis* detected from a flea pool in Luwero district matched to fleas collected from wild animals in Brazil (MH194356.1) and cat fleas from Brazil (MT012727.1) as well as ticks from Mexico and Slovakia respectively ON615337.1 and ON053295.1. The other sequence identified as *Rickettsia* spp. (next *felis*) by PCR was identical to *Candidatus* R. senegalensis sequences from a cat flea from Israel (MG893578.1) and *C. felis* from the United States (KU167051.1). Using the *ompA* gene, *R. felis* was detected in two *C. canis* pools from a goat and a dog, with one sequence confirmed by its *htrA* sequence. The *ompA* sequence comparison revealed *R. felis* from this study were identical to a sequence from a Brazilian flea (KY172883.1).

**Table 4.** Identity table comparing *Rickettsia* spp. sequences from this study to GenBank sequences by gene and district. The length of the *htrA* sequences were 326-425bp and *ompA* sequences were 545-612bp.

Gene	*Rickettsia* spp.	District	GenBank^a^	Accession ID	Identity
*htrA*	*R. asembonensis*	Luwero	OP974462	MK923739.1	100%
		Gulu	OP974452-OP974457, OP974463	KY445736.1	100%
		Kampala	OP974449-OP974451	KY445736.1	99.0%
		Kasese	OP974461, OP974467-OP974468, OP974470	MK923739.1	100%
		Jinja	OP974458-OP974460, OP974464-OP974466	KY445736.1	100%
	*R. felis*	Luwero	OP974469	MT012727.1^b^	100%
	*Rickettsia* spp.	Luwero	OP974471	MT012728.1	100%
*ompA*	*R. felis*	Luwero	OP985656, OP985657	KY172883.1^c^	100%

^a^Sequences from this study submitted to GenBank.

## Discussion

The main objective of this study was to detect emerging flea-borne pathogens and to describe flea-host associations in four regions of Uganda outside a plague-endemic area. Understanding flea-host associations and the pathogens they carry is critical in determining site specific outbreak potential of flea-borne febrile illnesses, which in turn can be used to guide prevention and control. Although there are surveys on fleas of small mammals in plague endemic areas and *Tunga* fleas in Busoga and Karamoja regions of Uganda [19,38,39] there are no comprehensive surveys of fleas parasitizing domestic animals in Uganda. We therefore report the diversity of flea species on livestock, pets, rodents, and environment in human dwellings in Uganda.

*C. canis* was the most abundant flea species (84.8%), parasitizing dogs, goats, rabbits, pigs, cattle, sheep, and cats, as well as from the dry soil environment in homesteads. This flea occurs worldwide and is the predominant flea found on dogs in Greece [40], Ireland [41], Chile [42], Albania [43] and Hungary [44] among other countries. However, our findings differ from others that found *C. felis* as the dominant flea on both dogs and cats in parts of sub-Saharan Africa [39,45]. Cat fleas, the second most abundant flea on domestic livestock and pets in our study are vectors of several zoonotic bacteria, including *Bartonella* spp., *Yersinia* spp., and *R. felis* [46,47]. The flea diversity on animals in close contact with humans is an important factor in predicting potential emergence of such zoonotic diseases. Animals that graze in the bush or hunt in the wild such as cats and dogs are likely to bridge the gap of circulation of certain diseases in the sylvatic cycle and domestic environments [48]. In our study, we identified the rodent flea *X. cheopis* on domestic cats and dogs which could increase the potential risk of spillover to humans.

Ecological factors, human-livestock interactions, and synanthropic pests (rats and mice) affect the flea population and diversity in an area [14,15]. These dynamic changes in host type and flea diversity are associated with the shift in foci of zoonotic diseases. For example, the epidemiologic focus for plague in the 1930s was in the southern portion of Uganda but shifted in the early 2000s to the current west Nile region where there are persistent outbreaks [49]. As a result of this change, small mammal populations have also adapted [19,49,50]. During intensive sampling of small mammals and their fleas in plague foci of southern and West Nile regions in 1937–1938, only one *R. rattus* was caught in the West Nile region. *Mastomys natalensis* was the most abundant rodent in human dwellings while *X. cheopis* was the most abundant flea [49]. But now, *R. rattus* is the most abundant rodent in human dwellings in the West Nile region with the same predominant flea, *X. cheopis* [19]. This agrees with our study, where *R. rattus* accounted for 74% of all rodent collections in human dwellings in all the five districts mainly infested with *X. cheopis*. Moreover, the specific flea index for *X. cheopis* (1.9) in this study was higher than 1.0. In areas where *X. cheopis* is the primary vector of *Y. pestis*, the risk of epizootic spread and exposure to humans increases when the specific flea index is greater than one [27]. This suggests that humans in those localities would be at risk if the bacteria is transmitted to humans.

*Rickettsia*-positive fleas were detected from every district, with varying MLEs. Higher MLEs and pool-positivity rates were in the Gulu district (northern Uganda) and Jinja district (eastern Uganda) suggesting potential hotspots for *Rickettsia* spp., warranting further investigation. Our study found a low pool positivity rate of *R. felis* in *C. canis*. *R. felis* is regarded as an emerging human pathogen associated with flea-borne spotted fever, a febrile illness largely neglected in routine public health surveillance in sub-Saharan Africa [51]. Despite the wide recognition of *R. felis* as a pathogen by several authors, there is still a controversy that needs critical review as to whether this organism is an obligate pathogen or opportunistic invader [52]. Although *C. felis* is a known competent vector for *R. felis* [53], the presence of this bacterium in *C. canis* poses a potential risk to human health however this is controversial due to its relative abundance and poor host specificity [52]. Moreover, *R. felis* transmission to humans has been associated with *Anopheles* mosquitos [54] and its DNA has been previously detected in other flea species [55]. Furthermore, *R. felis* has been identified in febrile patients in East Africa [51] and should be considered in diagnostic testing of febrile illness in these regions of Uganda. The pathogenicity of a *R. felis-*like organism, *Candidatus* R. senegalensis, is unknown [56,57]. In this study, *Candidatus* R. senegalensis was identified through phylogenetic analysis of the *htrA* gene and clustered with other sequences from cat fleas from Israel and the United States. While previously detected in Senegal, this is the first detection of *Candidatus* R. senegalensis in Uganda. The detection of *Candidatus* R. senegalensis from flea pools in Brazil was around 0.7%, which is lower than this study of 4% [58].

*R. asembonensis* belongs to the *R. felis*-like genotype group and its pathogen potential remains unknown. First detected in Asembo (Siaya County, Western Kenya) it was initially named as *R. asemboensis* [9], it has since been recorded in other parts of Africa, including Uganda [59] and South Africa [60]. The organism has also been found in fleas infesting domestic and wild animals in central and South America and our *htrA* sequences are highly identical which could be explained by this highly conserved region [17,61]. The predominant species identified by sequencing in our study was R. *asembonensis*, which constituted 92% (23/25) of the species identified. This trend has been observed in Kenya where the agent was first detected and *R. felis* formed less than 10% of the rickettsia detected in fleas [18]. Further to west of Uganda, *R. asembonensis* was identified as the sole species in fleas from dogs in Rwanda [47]. This pattern suggests *R. asembonensis* as the likely predominant flea-borne *Rickettsia* in the East African region.
*Y. pestis* was not detected in any flea pool, which still warrants reporting, given repeated plague outbreaks in neighboring West Nile region [13,24]. *Y. pestis* often causes epidemics with high case fatality rates in untreated patients drawing public health attention [13]. Even within plague endemic areas in Uganda, rodent fleas in quiescent plague periods had no *Y. pestis* detected [62]. Our finding agrees with the lack of human cases in other regions of Uganda despite a long history of cases in the West Nile region.

## Limitations

Flea pools were solely identified morphologically, potentially misclassifying flea species. By pooling fleas, the MLE was immeasurable when 100% of flea pools were positive and MIR was immeasurable when 0% of flea pools were positive and this was noted in Table 3 using dashes (-). Pooling of fleas, though increases the pathogen detection rate, it however has limitation of discriminating mixed infections and so, co-infection was not assessed.

## Conclusion

We confirm the presence of *R. felis* in Luwero district, which suggests a potential risk of human infection. We also demonstrate the wide distribution of *R. asembonensis* as the predominant *Rickettsia* spp. in multiple flea species in the four regions of Uganda. Given that these animals were collected near homesteads, there is a risk for human exposure, with future efforts expanding differential diagnosis to include *R. felis* and *R. asembonensis* in acute febrile patients with unknown etiology. As the first detection of *Rickettsia* spp. in *E. gallinacea*, expanded monitoring of homesteads in other areas of Uganda would contribute to the characterization of potential flea-borne disease threats and causes of febrile illness throughout the country. While we did not detect *Y. pestis*, the wide distribution of competent vectors across study sites is of concern, should *Y. pestis* be introduced to these districts. Further studies are required to determine the full extent of *Rickettsia* spp. and *Y. pestis* in these Ugandan districts.

## Supplementary Material

Supplementary File 1.docx

Supplementary File 2.docx

## Data Availability

The datasets used or analyzed in the current study are available with the corresponding author on reasonable request. The sequences from this study are available on GenBank under the accession numbers: OP974449-OP974471. The tables and figures are incorporated in the manuscript. Further supplementary files are attached as a separate file.

## Abbreviations

PCR: Polymerase Chain Reaction
qPCR: Quantitative realtime PCR
*gltA*: Citrate synthase gene
*htrA*: Gene encoding for outer membrane protein with molecular mass of 17 kilo Daltons
*ompA*: Outer membrane protein A gene
MIR: Minimum infection rate
MLE: Maximum likelihood estimate
FBSF: Flea-borne spotted fever
