# Supplementary material for: Molecular detection and characterization of Rickettsia felis, R. asembonensis, and Yersinia pestis from peri-domestic fleas in Uganda
Source: Infect Ecol Epidemiol. 2025 Mar 3;15(1):2473159. doi: 10.1080/20008686.2025.2473159 (PMC11878166; doi:10.1080/20008686.2025.2473159)
Supplement: Supplementary File 1.docx [file ZIEE_A_2473159_SM2752.docx]

Supplementary File 1

Table 1: Number of rodents captured in different quarters (seasons)

| Districts | Rat species | Numbers captured in different quarters (seasons) | | | | | | Total | Percentage abundance |
| --- | --- | --- | --- | --- | --- | --- | --- | --- | --- |
|  |  | 2017 | | | 2018 | | |  |  |
|  |  | Apr-Jun^a^ | Jul-Sep^b^ | Oct-Dec^a^ | Jan-Mar^b^ | Apr-Jun^a^ | Jul-Sep^b^ |  |  |
| Kampala | *R.rattus* | 8 | 2 | 4 | 5 | 4 | 2 | 25 | 58.1 |
|  | Shrew | 1 | 0 | 0 | 0 | 1 | 0 | 2 | 4.7 |
|  | *M. musculus* | 1 | 3 | 4 | 2 | 4 | 2 | 16 | 37.2 |
| Kasese | *R.rattus* | 11 | 5 | 7 | 9 | 8 | 4 | 44 | 91.7 |
|  | Shrew | 1 | 0 | 0 | 0 | 0 | 0 | 1 | 2.1 |
|  | *M. musculus* | 0 | 0 | 1 | 0 | 0 | 2 | 3 | 6.2 |
| Jinja | *R.rattus* | 8 | 2 | 2 | 1 | 5 | 0 | 18 | 64.3 |
|  | Shrew | 0 | 0 | 0 | 0 | 2 | 1 | 3 | 11.5 |
|  | *M. musculus* | 2 | 0 | 1 | 0 | 2 | 0 | 5 | 19.2 |
| Luwero | *R.rattus* | 4 | 1 | 2 | 1 | 1 | 0 | 9 | 60 |
|  | Shrew | 0 | 0 | 0 | 1 | 0 | 0 | 1 | 6.7 |
|  | *M. musculus* | 0 | 0 | 0 | 1 | 2 | 2 | 5 | 33.3 |
| Gulu | *R.rattus* | 5 | 4 | 4 | 2 | 2 | 5 | 22 | 81.5 |
|  | Shrew | 0 | 0 | 1 | 0 | 1 | 0 | 2 | 7.4 |
|  | *M. musculus* | 0 | 0 | 0 | 0 | 1 | 2 | 3 | 11.1 |
| Total number of rodents captured per quarterly season | | 41 | 17 | 26 | 22 | 33 | 20 | 159 |  |

^a^Rainy season

^b^Dry season

Table 2: Total Number of animals examined per quarter (season). The animals listed here are those that were able to be restrained and possible to comb the fur for collection of fleas.

| Districts | Animal species | Numbers of animals examined in different quarters (seasons) | | | | | |  |
| --- | --- | --- | --- | --- | --- | --- | --- | --- |
|  |  | 2017 | | | 2018 | | |  |
|  |  | Apr-Jun^a^ | Jul-Sep^b^ | Oct-Dec^a^ | Jan-Mar^b^ | Apr-Jun^a^ | Jul-Sep^b^ | Total |
| Kampala | Cat | 4 | 3 | 6 | 5 | 4 | 9 | 31 |
|  | Dog | 12 | 13 | 10 | 11 | 14 | 21 | 81 |
|  | Goat | 32 | 29 | 32 | 28 | 36 | 38 | 195 |
|  | Rabbit | 0 | 0 | 2 | 0 | 0 | 0 | 2 |
| Kasese | Cat | 5 | 4 | 5 | 6 | 3 | 4 | 27 |
|  | Dog | 11 | 9 | 10 | 9 | 10 | 12 | 61 |
|  | Goat | 46 | 43 | 44 | 44 | 45 | 42 | 264 |
|  | Rabbit | 0 | 0 | 0 | 0 | 2 | 3 | 5 |
| Jinja | Cat | 3 | 2 | 6 | 5 | 6 | 2 | 24 |
|  | Dog | 11 | 12 | 9 | 12 | 13 | 12 | 69 |
|  | Goat | 43 | 42 | 40 | 46 | 43 | 44 | 258 |
|  | Rabbit | 0 | 0 | 0 | 0 | 0 | 0 | 0 |
| Luwero | Cat | 3 | 3 | 3 | 4 | 3 | 2 | 18 |
|  | Dog | 10 | 11 | 12 | 10 | 11 | 12 | 66 |
|  | Goat | 33 | 28 | 38 | 36 | 35 | 36 | 206 |
|  | Rabbit | 2 | 0 | 1 | 0 | 0 | 0 | 3 |
| Gulu | Cat | 2 | 3 | 7 | 4 | 2 | 4 | 22 |
|  | Dog | 12 | 10 | 12 | 11 | 9 | 12 | 66 |
|  | Goat | 48 | 46 | 43 | 45 | 45 | 46 | 273 |
|  | Rabbit | 3 | 0 | 0 | 0 | 0 | 0 | 3 |
| Total number of animals examined per quarterly season | | 280 | 258 | 280 | 276 | 281 | 299 | 1674 |

^a^Rainy season

^b^Dry season
