# Supplementary material for: Molecular detection and characterization of Rickettsia felis, R. asembonensis, and Yersinia pestis from peri-domestic fleas in Uganda
Source: Infect Ecol Epidemiol. 2025 Mar 3;15(1):2473159. doi: 10.1080/20008686.2025.2473159 (PMC11878166; doi:10.1080/20008686.2025.2473159)
Supplement: Supplementary File 2.docx [file ZIEE_A_2473159_SM2751.docx]

Supplementary File 2

Table 1: Individual fleas (pools) collected by quarters (seasons)

| Quarter | *Fleas collected (Flea pools)* | | | | | |  |
| --- | --- | --- | --- | --- | --- | --- | --- |
|  | *C. canis* | *C. felis* | *E. gallinacean* | *P. irritans* | *X. cheopis* | Total | |
| 1 (Apr-Jun, 2017)^a^ | 345(48) | 8(2) | 66(10) | 1(1) | 72(20) | 492(81) | |
| 2 (Jul-Sep, 2017)^b^ | 1211(61) | 63(8) | 659(12) | 0(0) | 32(11) | 1965(92) | |
| 3 (Oct-Dec, 2017)^a^ | 2208(81) | 91(13) | 156(18) | 0(0) | 29(11) | 2484(123) | |
| 4 (Jan-Mar, 2018)^b^ | 2164(70) | 243(15) | 91(11) | 0(0) | 23(8) | 2521(104) | |
| 5 (Apr-Jun, 2018)^a^ | 2318(96) | 51(10) | 256(14) | 0(0) | 49(10) | 2674(130) | |
| 6 (Jul-Sep, 2018)^b^ | 4176(145) | 148(13) | 128(8) | 0(0) | 53(18) | 4505(184) | |
| Total | 12422(501) | 604(61) | 1356(73) | 1(0) | 258(78) | 14641(714) | |

^a^Rainy season

^b^Dry season

Table 2: Individual fleas (pools) collected by district

| District | *Fleas collected (Flea pools)* | | | | | |
| --- | --- | --- | --- | --- | --- | --- |
|  | *C. canis* | *C. felis* | *E. gallinacea* | *P. irritans* | *X. cheopis* | Total |
| Gulu | 1960(96) | 88(13) | 20(5) | 1(1) | 44(13) | 2113(128) |
| Jinja | 2501(105) | 223(14) | 270(14) | 0(0) | 51(14) | 3045(147) |
| Kampala | 2127(83) | 122(13) | 152(7) | 0(0) | 56(20) | 2457(123) |
| Kasese | 2786(109) | 74(13) | 540(31) | 0(0) | 93(27) | 3493(180) |
| Luwero | 3048(108) | 97(8) | 374(16) | 0(0) | 14(4) | 3533(136) |
| Total | 12422(501) | 604(61) | 1356(73) | 1(0) | 258(78) | 14641(714) |
